# How do steppe plants follow their optimal environmental conditions or persist under suboptimal conditions? The differing strategies of annuals and perennials

**DOI:** 10.1002/ece3.3664

**Published:** 2017-11-23

**Authors:** Hocine Ait Mouheb, Leila Kadik, Cécile Hélène Albert, Rachda Berrached, Andreas Prinzing

**Affiliations:** ^1^ Laboratory of Ecology and Environment Faculty of Biological Sciences University of Sciences and Technology Houari Boumediene Bab Ezzouar Algiers Algeria; ^2^ CNRS IRD IMBE Europôle Méditerranéen de l'Arbois Aix Marseille Univ Univ Avignon Aix‐en‐Provence Cedex 04 France; ^3^ Research Unit “Ecosystèmes Biodiversité, Evolution” Centre National de la Recherche Scientifique University Rennes 1 Rennes France

**Keywords:** false‐negative rate, false‐positive rate, following optimal environmental conditions, functional traits, persisting under suboptimal environmental conditions, species distribution model

## Abstract

For a species to be able to respond to environmental change, it must either succeed in following its optimal environmental conditions or in persisting under suboptimal conditions, but we know very little about what controls these capacities. We parameterized species distribution models (SDMs) for 135 plant species from the Algerian steppes. We interpreted low false‐positive rates as reflecting a high capacity to follow optimal environmental conditions and high false‐negative rates as a high capacity to persist under suboptimal environmental conditions. We also measured functional traits in the field and built a unique plant trait database for the North‐African steppe. For both perennial and annual species, we explored how these two capacities can be explained by species traits and whether relevant trait values reflect species strategies or biases in SDMs. We found low false‐positive rates in species with small seeds, flowers attracting specialist pollinators, and specialized distributions (among annuals and perennials), low root:shoot ratios, wide root‐systems, and large leaves (perennials only) (*R* ^*2*^ **= .52–58). We found high false‐negative rates in species with marginal environmental distribution (among annuals and perennials), small seeds, relatively deep roots, and specialized distributions (annuals) or large leaves, wide root‐systems, and monocarpic life cycle (perennials) (*R* ^*2*^ **= .38 for annuals and 0.65 for perennials). Overall, relevant traits are rarely indicative of the possible biases of SDMs, but rather reflect the species' reproductive strategy, dispersal ability, stress tolerance, and pollination strategies. Our results suggest that wide undirected dispersal in annual species and efficient resource acquisition in perennial species favor both capacities, whereas short life spans in perennial species favor persistence in suboptimal environmental conditions and flowers attracting specialist pollinators in perennial and annual species favor following optimal environmental conditions. Species that neither follow nor persist will be at risk under future environmental change.

## 1 INTRODUCTION

In the last century, environmental conditions have changed at a rate that is unprecedented in the recent history of life, and species have to respond to these changes (Jackson & Overpeck, 2000). Some species may survive under modified environmental conditions, while others may succeed in tracking the spatial shift of their optimal environmental conditions (“optimal” is here pragmatically inferred from the environmental conditions in which the species usually occurs). Finally, some species may successfully use both strategies; others will fail and face extinction (Chevin, Lande, & Mace, 2010). Among plant species, all these scenarios have already been observed under past environmental changes(Van der Putten, Macel, & Visser, 2010), but we still know very little about why species differ in their capacity to follow shifts in their optimal conditions (i.e., conditions of maximal growth, minimal stress for species (Keddy, 2017), environmental conditions, or to persist under suboptimal conditions (i.e., conditions of reduced growth due to increased stress for species (Keddy, 2017)). This raises the question of whether or not there are particular traits that favor either of these capacities.

There are a number of hypotheses to investigate (Table 1). It might be the case that both capacities, following optimal environmental conditions and persisting under suboptimal conditions, depend on the same set of trait values, notably those that increase capacity to disperse, establish individuals, and maintain populations (Ghedini & Southern, 2015) (Table 1 I). Specifically, high dispersal capacity will decrease dispersal limitation and may result from morphological dispersal syndromes permitting anemochorous and zoochorous dispersal across large distances (Ozinga et al., 2005), and by large plant height increasing the diameter of the seed shadow (Estrada et al., 2015; Vittoz & Engler, 2007). Moreover, dispersal capacity may be increased by light seeds, which are more easily transported by biotic and abiotic dispersal agents (Khurana, Sagar, & Singh, 2006). Once dispersed, individuals need to maintain themselves and tolerate local environmental conditions. Tolerance to drought, for instance, may be conferred by a deep or wide root‐system permitting access to deep or distant soil water (Pouget, 1979), or by trait values limiting water loss, such as small specific leaf area or high leaf dry‐matter content (LDMC) (Lopez‐Iglesias, Villar, & Poorter, 2014). Tolerance to drought might also be conferred by extensive dormant stages such as long seed dormancy, notably in annual species (Aidoud, 1997). Established individuals also may need to be competitive, that is, to efficiently acquire resources, which may be favored by trait values such as large leaf area, or high specific leaf area and a high stature (e.g., in dry regions: chamaephyte “shrub” life form (Muller‐Landau, Wright, Calderón, Condit, & Hubbell, 2008). The competitiveness of seedlings may also increase with seed size (Estrada et al., 2015). Finally, the plant needs to ensure sufficient reproductive rates to maintain populations, which requires high pollination rates. Pollination rates may be increased by flower shapes facilitating wind pollination, or pollination via a wide range of pollinators such as dish‐shaped flowers (Rodriguez‐Gironés & Santamaria, 2010), or, alternatively, by flower shapes attracting specialized pollinators that come with the right, conspecific pollen, such as deep and flag‐shaped flowers (Fenster, Martén‐Rodríguez, & Marten‐Rodriguez, 2007).

**Table 1:** Hypotheses and predictions regarding the relationships between traits and false‐positive and false‐negative rates. In bold, predictions that were confirmed, and in italic, predictions that were contradicted by the results presented in this study for annuals (^(a)^) or perennials (^(p)^)

	A species occurs often where one does not expect it (many false negatives)	A species occurs everywhere where one expects it (few false positives)
I. Capacities of species to follow their optimal environmental conditions and to persist under suboptimal conditions depend on the same trait values		
Accessing new localities and establishing new populations	**Long distance dispersal, that is, small seeds** ^**(a)**^, wind/animal dispersed	
* *Maintaining established individuals	Tolerating abiotic harshness, that is, deep roots, small SLA, high LDMC, relatively small above‐ground body, **wide root system** ^**(p)**^Competitive superiority due to efficient resource acquisition, that is, high SLA, **large leaf area** ^**(p)**^	
* *Maintaining seeds and seedlings	Persist across unfavorable periods by dormant stage, that is, annuals, or gain competitive advantage by large seeds	
* *Maintaining local populations	Not depending on specialized pollinators that might be absent, or interact efficiently with specialized pollinators	
II. Capacities of species to follow their optimal environmental conditions and to persist under suboptimal conditions depend on different trait values		
* *Accessing new localities and establishing new populations	Large numbers of seeds, **undirected dispersal, that is, small seeds** ^**(a)**^ or seeds with particular adaptation for wind dispersals	Directed dispersal, for example, by animals
* *Maintaining established individuals	…under the abiotically harsh conditions found in suboptimal environment, that is, deep roots, small SLA, high LDMC, **relatively high shoot:root ratio** ^**(a)**^ **, wide root system** ^**(p)**^	…under abiotically favorable conditions: competitive superiority due to efficient resource acquisition, that is, high SLA, **relatively low shoot: root ratios** ^**(p)**^
* *Maintaining seeds and seedlings	… in only temporally suitable environment: dormant stage, that is, annuals, **monocarpic perennials** ^**(p)**^	… in permanently suitable environment: *gaining competitive advantage by large seeds* ^(p)^
* *Maintaining local populations	…in a new community neighborhood: not depending on specialized pollinators as they might be absent	…in a known established community neighborhood: **interact efficiently with specialized pollinators** (confirmed for gullet^(a)^ and flag^(p)^ shaped flowers, but not for dish‐shaped^(p)^)
III. Environmental fluctuations result in spatial mismatch between species distribution and their optimal environmental conditions		
* *Delay in responding to environmental fluctuations	*Long delay in long‐lived or chamaephyte (perennial)species increasing mismatch between species distributions and the environment* ^(p)^	Short delay in short‐lived or annual species decreasing mismatch^(p)^
IV. Methodological shortcomings and sampling		
* *Model quality	Many false predictions if species range is largely outside study area, that is, species ecologically marginal	Species ecologically central
* *Occupied *vs*. available environmental conditions	Many still unoccupied environmental conditions available for species of or **specialized distribution** ^**(a)**^	
* *Detectability of species		Species permanently present above‐ground are not overlooked, that is, chamaephytes, or species with relatively high above‐ground body

SLA, specific leaf area; LDMC, leaf dry‐matter content, (a) = annual, (p) = perennial, (t) = annual and perennial.

A further hypothesis is that the trait values required to follow optimal environmental conditions might differ from those required to survive in suboptimal conditions, resulting in a possible trade‐off between investment in either capacity (Aubin et al., 2016) (Table 1 II). For instance, following optimal environmental conditions might require directed dispersal (e.g., through animals) and local competitive dominance through efficient resource acquisition (e.g., high specific leaf area or high leaf area), large seeds, and large stature. Optimal environmental conditions might also enable efficient interactions with reliably available specialized pollinators, for example, through specialized flowers (Castro‐Urgal & Traveset, 2016). In contrast, persisting under suboptimal environmental conditions might require accessing a wide range of sites through the undirected dispersal of small seeds by the wind, and tolerating particularly extreme abiotic conditions through reduced water loss (e.g., small specific leaf area) and efficient water acquisition (e.g., deep roots and wide root‐system) (Burns, 2004; Liu et al., 2014; Muller‐Landau et al., 2008; Padilla & Pugnaire, 2007). Persisting in suboptimal environmental conditions might also be favored by dish‐shaped, shallow flowers avoiding dependency on specialized interactions with pollinators as these pollinators might be unavailable (Castro‐Urgal & Traveset, 2016).

We can also hypothesize that following optimal environmental conditions is favored by more stable environmental conditions, while high environmental variability may lead to a mismatch between the environmental conditions and species distributions (Michel & Knouft, 2012). A given environment might be stable from the point of view of an annual species, but fluctuating from the point of view of a perennial species (Table 1 III). We may therefore hypothesize that long‐lived species could more easily persist under suboptimal environmental conditions than short‐lived species.

The hypotheses developed above provide testable predictions on the strength and the nature of the relationship between species trait values and their capacity to follow their optimal environmental conditions or to persist under suboptimal conditions (see Table 1). To test these predictions, we propose confronting field‐measured trait values with the predictive performance of species distribution models (SDMs). Indeed, a species which is present on most sites predicted to be suitable (low false‐positive rates) is likely to have succeeded in following its optimal environmental conditions wherever they occur (Michel & Knouft, 2012). A species which is often present on sites that are predicted to be unsuitable (high false‐negative rates) is likely to have succeeded in persisting under suboptimal environmental conditions (Hanspach, Kühn, Schweiger, Pompe, & Klotz, 2011). This approach supposes that false negatives represent viable populations that persist in a suboptimal environment. We admit that this is not necessarily the case; false negatives might be population sinks. We note that if false negatives were sink populations, we should not expect any link between the false‐negative rate and traits that indicate particular capacities of species, except for the capacity of undirected dispersal. In the present study, we did find numerous such relationships. Nevertheless, we cannot rule out that false negatives are in part sink populations.

It should be noted that ranking species' capacity to follow their optimal environmental conditions or to persist in suboptimal conditions might be influenced by methodology and sampling (Table 1 IV). The available data and sampling may strongly influence our ability to identify a species' optimal environmental conditions and hence the species' ability to follow its optimal conditions or to persist under suboptimal conditions. Inevitably, any statistical approach—such as SDMs—assumes the equilibrium of species with their environment, and that the study area accurately encompasses the species' niche (Franklin, 2010). Departing from these assumptions might lead to under‐ or overestimates of the suitability of a given local environment for a given species (Guisan & Thuiller, 2005). In particular, estimates of the optimal environmental conditions will be the least accurate in species, which occupy environmental conditions that are ecologically marginal to the study area, that is, which the study area does not accurately represent. Another methodological problem is the poor detectability of species leading to the apparent absence of some species in optimal environmental conditions. Detectability can be particularly low in species with short shoots and annual life forms or life forms that temporally retreat belowground. In addition, the species' degree of specialization may be biologically important, but it also introduces a methodological bias: For a specialist species, there may be numerous suboptimal sites. This increases the likelihood of finding the species in question by chance in some of these numerous suboptimal sites (Hanspach et al., 2011). Overall, just like the different biological mechanisms, the different methodological limitations also provide testable predictions on the relationship between trait values and species' capacity to follow their optimal environmental conditions or to persist under suboptimal conditions.

While multiple studies have linked range size to traits (Dobrowski et al., 2011; Guisan et al., 2007; Hanspach, Kühn, Pompe, & Klotz, 2010; Pöyry, Luoto, Heikkinen, & Saarinen, 2008; Soininen & Luoto, 2014), few tried to understand which species traits improve the capacity to follow their optimal environments or to persist under suboptimal ones. The few existing ones have not used quantitative eco‐morphological traits (Guisan et al., 2007) and in particular did not consider root traits which we may find to be major importance in dry environments. Here, we suggest closing this gap. We propose confronting field‐measured trait values with the predictive performance of species distribution models on a set of 135 steppe plant species. Steppe plants are known to be particularly vulnerable to environmental change, as they may suffer from water deficit and other harsh climatic conditions (Pouget, 1979). We developed fine‐grained SDMs based on information on local occurrences and environmental conditions (Guisan & Thuiller, 2005) and quantified the predictive performance of these SDMs for each species. We then correlated rates of false‐positive and false‐negative predictions to a set of functional traits and niche traits (marginality and specialization of species environmental distributions). Given that most of the species studied had not already been recorded in global trait databases, the trait values used in this study were measured in the field or in the lab for the purposes of this study, thus resulting in what might be the first large database of functional traits for Northern African steppe species. We asked three questions: (i) How can species capacities to follow their optimal environmental conditions or to persist under suboptimal conditions be explained by trait values reflecting reproductive strategy, dispersal ability, stress tolerance, and pollination strategies? (ii) Do different trait values affect these two capacities differently? (iii) Do relevant trait values reflect species strategies or methodological biases? We conducted analyses separately for annuals and for perennials as the two groups differ fundamentally in their ability to establish the above‐mentioned traits and might hence employ different strategies to follow optimal and persist in suboptimal environments.

## 2 MATERIALS AND METHODS

### 2.1 Study area and vegetation survey

Our study area was located in Southern Algeria and encompasses the Algerian steppes that extend from the Tellian Mountains in the north to the vast Saharan areas in the south. The study area extends from 2° to 4° Eastern longitude and from 34° to 36° Northern latitude (Figure S1). The steppe covers a wide range of environmental conditions, as there is a gradual transition from a sub‐Mediterranean climate in the north to a comparatively more desert‐like climate in the south (Pacini & Nicolson, 2007).

We used plant distribution data from database “sol‐vegetation‐Algeria,” which contains 1210 phytosociological plots recorded between 1968 and 1975 across a grid at 1 km² resolution, covering 350 species (see Figure S1). From this database, we selected 832 plots which covering most of the steppes of Southern Algeria as the study region (except forest vegetation). The selected database contains comprehensive surveys of vegetation with real presences/absences for the 135 species that occurred in more than 30 plots and with abundance greater than 5% to ensure the robustness of the calibrated SDMs.

### 2.2 Selected traits

We chose 14 traits known to be related to species' capacity to tolerate harsh environmental conditions, to disperse, to establish new populations, to proliferate, and related to habitat specificity. The information on how each trait was measured and categorized is set out below. These traits were measured in the field in 2015 on five to 25 individuals per species. Measured individuals were randomly selected within our plots spatially scattered along the environmental gradient of our study area and from reproductively mature, healthy‐looking specimens with no severe damage from herbivores or pathogens. The mean values calculated across the sampled plants of a given species are likely representative of the traits across the entire species, although we have no objective way of measuring the representativeness of our means. Any remaining deviation from the real means are likely random, rather than biased with respect to false positives or negatives from our niche models.

Seed (dry) mass, expressed in mg, was measured for 63 species by harvesting seeds from five individuals. For large‐seeded species, 50 seeds per individual were weighed, and for small‐seeded species, 100–200 seeds per individual were weighed. Seeds were placed in glass vials and dried overnight at 80°C, and they were then put into a heat chamber and subsequently weighed on precision scales (Pérez‐Harguindeguy et al., 2013).

Dispersal modes were classified for each of the initial 135 species: We identified the principal dispersal mode based on seed morphology and how it relates to one of the three dispersal vectors (using criteria given in (Vittoz & Engler, 2007): zoochory, anemochory, autochory).

*Root depth and width of root‐system* are two important traits related to species draught‐tolerance. They were measured on five individuals per species for 84 different species. For root depth, we measured the maximum rooting depth, and for the width of the root‐system, we measured the distance from the center of plant to the furthest lateral root of that plant, expressed in meters (Pérez‐Harguindeguy et al., 2013).

*Plant height* is related to (low) tolerance of climatic extremes and (high) competitiveness (Rodríguez‐Gironés & Santamaría, 2007). We measured the distance between the upper boundary of the main photosynthetic tissues of a plant and ground level for 25 mature individuals per species (measured on 84 species), expressed in meters (Pérez‐Harguindeguy et al., 2013).

*Root:shoot ratio* is the ratio of the two previous traits, that is, the relative investment in either function.

*Life span* was classified for all 135 species based on “New flora of Algeria” (Quezel & Santa, 1962, 1963) as “annuals” are plants with a life cycle that lasts only 1 year, while “perennials” are plants that lives more than 1 year.

*Life form* was classified for all 135 species into four categories based on the position of the floral shoots relative to ground level (Kadik, 2012; Raunkiær, 1977): therophytes, geophyte, hemi‐cryptophyte, and chamaephyte; phanerophytes being absent from the steppe by definition (Raunkiær, 1977). We added the category of monocarpic (or “semelparous”), that is, species that reproduce once and then die, as these species often have a shorter life span than other perennials (Pérez‐Harguindeguy et al., 2013).

Leaf area (LA), specific leaf area (SLA), and leaf dry‐matter content (LDMC) were measured for 84 species based on 15 mature leaves harvested from five different adult individuals per species, following the protocol established by Rodríguez‐Gironés et al. (Torres & Galetto, 2002). The fresh leaves were cut from the stem, weighed, and scanned. Subsequently, we measured each leaf's surface area in mm² using the software program ImageJ (Image processing and analysis in Java). Leaves were then dried at 70°C for at least 72 hr before being weighed again. To determine SLA, we divided the one‐sided area of a fresh leaf (mm²) by its oven‐dry mass (mg). For LDMC, we divided the oven‐dry mass (mg) of a leaf, by its water‐saturated fresh mass (g).

Marginality and tolerance of environmental distribution were quantified using ecological niche factor analysis (ENFA) to characterize each species' niche (Hirzel et al. 2002). The marginality of a species is defined as the difference between the global mean of the variables and the species mean, normalized by dividing by 1.96 standard deviation (Hirzel et al. 2002). Marginality close to “0” indicates that the species tends to live in average environmental conditions, while a high value (close to 1) indicates a tendency to live in extreme habitats. The tolerance of a species is defined as the ratio of the standard deviation of the global distribution to that of the focal species (Hirzel et al. 2002). A tolerance close to “0” indicates a specialist species tending to live in a very narrow range of conditions, while a high value (close to 1) indicates a generalist species. To ensure consistency, we quantified distributions of species based on the same environmental predictors (climate, soil, land use) as those used for the SDMs (see below).

*Corolla flower depth* is related to the length of the proboscis of the insect visitors and nectar concentration (Pacini & Nicolson, 2007; Rodríguez‐Gironés & Santamaría, 2007). Deeper flowers tend to attract more specialized pollinators (Aubin et al., 2016; Castro‐Urgal & Traveset, 2016). We measured the distance between corolla insertion and the beginning of corolla lobes for one flower from each of five individuals per species (measured on 66 species) using an electronic caliper (resolution = 0.01 mm) (Torres & Galetto, 2002). Species with no flower tube (e.g., *Aizoon hispanicum* L*, Herniaria fontanesii J. Gay*) were ranked as zero.

*Flower shape* was classified for 84 species, following those defined by (Faegri & van der Pijl, 1966): dish‐shaped, bell‐shaped, tube‐shaped, flag‐shaped, gullet‐shaped, or without obvious floral attractants (Nicolson & Thornburg, 2007). Bell‐shaped species were later removed for the analyses because there were only two species in this category.

Niche trait values were measured for all traits for 62 species.

### 2.3 Environmental predictors

We used four types of environmental variables that are expected to be strong determinants of the distribution of steppe plant species. We used integrative variables as they permit capturing much variation with a still manageable number of variables. 1) We used three climate variables, averaged for the period 1950–1990, namely mean annual precipitation, mean minimal January temperature, and mean maximal July temperature at a resolution of 1 × 1 km (Worldclim (Hijmans, Cameron, Parra, Jones, & Jarvis, 2005)). 2) We used the topographic moisture index (Syphard & Franklin, 2010) that expresses relative humidity and was calculated from a digital elevation model from which we calculated the upslope catchment area and slope angle at a resolution of 1 × 1 km. 3) We used soil type: coarse mineral soils, poorly developed soils, isohumic soils, halomorphic soils, hydromorphic soils, or calci‐magnesian soils (soil map of Algeria, scale of 1/500,000 (Durand, 1953; ). 4) We used soil occupation: forest and matorral, alfa (*Stipa tenacissima*. L) steppes, chamaephyte steppes, halophyte steppes, crops, and urban. The soil occupation map of the south Algiers steppe (resolution 1 × 1 km) (Sehl & Guettouche, 2015) is based on classifications by maximum likelihood of Landsat MSS 1972 images.

### 2.4 Modeling species distribution

We used the database “sol‐vegetation‐Algeria,” which contains comprehensive surveys of vegetation with real presences/absences for the 135 species that we related with environmental variables, and we parameterized species distribution models using the biomod2 (Thuiller, 2003; Thuiller, Lafourcade, Engler, & Araújo, 2009) library in the statistical programming environment “R” (3.2.3). We ran all the model types: two regression methods (generalized linear models, GLM; and generalized additive models, GAM), two machine learning methods (artificial neural networks, ANN; random forest, RF), and three classification methods (factorial discriminant analysis, FDA; classification tree analysis, CTA; and generalized boosted models, GBM).

Models were parameterized with a random subset containing 75% of all plots. The remaining 25% were used for the validation of predictions. The random selection of a single set of modeling sites and a single set of prediction sites might introduce some noise, but not bias. Noise should prevent detection of relationships. However, we found that the prediction errors were highly correlated with species traits and hence conclude noise was likely no problem. The binary transformation of the outputs was carried out using the threshold that maximized the true skill statistics (TSS, (Allouche, Tsoar, & Kadmon, 2006). In order to assess the models' predictive capacity, we calculated the false‐negative rate, the false‐positive rate, the area under the ROC curve (AUC, (Swets, 1988), and the true skill statistics (Allouche et al., 2006). The AUC is comprised between 0 and 1; the closer it is to 1 the higher the predictive power (Swets, 1988). We chose to further consider only the results obtained with the GLMs because it was the only model with both high AUC (>0.70) and high TSS (>0.50) values for all species and applicable with a high number of species compared to either of the alternative models. Furthermore, the variance of predictive performances among species was greater in GLMs than in the alternative models. Thus, considering more models would likely result in a loss of power but otherwise not change the relationships between rates of prediction errors and species traits.

We interpreted low false‐positive rates as an indicator of a high capacity to follow optimal environmental conditions and high false‐negative rates as an indicator of a high capacity to persist under suboptimal environmental conditions.

### 2.5 Relating prediction errors to species trait values

We first investigated whether among‐species variation in the rates of false‐positive and false‐negative predictions could be explained by differences between annuals (25 species) and perennials (35 species), using a simple ANOVA.

We then explored the effect of species' evolutionary position on their trait values to account for their phylogenetic nonindependence (Ricklefs, Starck, & Rickfs, 2010). There is currently no phylogeny available for the flora of Algeria; we hence used taxonomy, at the family level, as a surrogate. The family level seemed appropriate because several of the measured traits have conserved values at this level (Prinzing, Durka, Klotz, & Brandl, 2001) and because we often had no replicate species within genera. We used simple ANOVAs to test whether families explained the differences in trait values. We found that a single family had a significant effect: Brassicaceae (four annual species and one perennial species) species showed particularly high false‐negative rates and particularly low false‐positive rates. However, “Brassicaceae” membership was strongly related to flower shape resulting in strong multicollinearity (tolerances < 10%) of both variables. We hence excluded “Brassicaceae” and kept flower shape noting that the two variables are difficult to separate.

Finally, we used ordinary least squared regressions to investigate whether among‐species variation in the false‐positive and false‐negative rates could be explained by species traits (root depth, width of root‐system, plant height, root: shoot ratio, seed mass, leaf area, specific leaf area, leaf dry‐matter content, dispersal modes, flower shape, and the marginality and tolerance of environmental distributions). The analyses were repeated for perennials and annuals separately; “Brassicaceae” and (for perennials) *life form* were also included as explanatory variables.

We used Mallow's Cp technique to select the best subset of nonredundant explanatory variables (Mallow's Cp maximizes explanatory power rather than minimizing numbers of variables; Everitt & Howell, 2005). Unfortunately, the remaining models still contained some explanatory variables highly correlated to the others, that is, more than 90% of the variance of these explanatory variables was explained by other variables (“tolerance” < 0.10). Hence, of two correlated variables, we eliminated the variable with the lowest tolerance in order to increase the tolerance of the variable that we have kept in the model. This procedure allows simultaneously keeping a maximum of explanatory variables and reducing multicollinearity and hence improving the quality of our models. The model does not contain interaction terms. Although biologically plausible, these terms would risk to result in over‐parametrization, and even without interaction terms explained variances were high.

We used QQ plots and predicted/residual to explore normality and homogeneity of residuals and log (10)‐transformed variables where needed. In order to better illustrate the results of general regression models, we calculated partial residuals (Tables S2–S5), that is, the residuals of false‐positive and false‐negative rates of perennial and annual species, accounting for the variance explained by all independent variables. For categorical variables, partial residuals were calculated separately for each category. The predicted versus residual plots showed two outlier species with very high false‐negative rates relative to other species: *Launaea nudicaulis* (L.) Hook.f and *Noaea mucronata* (Forsk.) Asch. et Schw. After excluding these two outliers, there were no further major outliers in the residuals' distribution.

All statistical analyses were performed using STATISTICA.10.

## 3 RESULTS

### 3.1 Characterizing species

We observed large variations in most trait values and in false‐positive and false‐negative rates (Table S1). Table 2 gives the 10 species (i) with particularly low rates of false positives, that is, capable of following their optimal environmental conditions, (ii) with particularly high rates of false negatives, that is, capable of persisting under suboptimal environment conditions, and (iii) presenting neither capacity.

**Table 2:** lists of species characterized by extreme rates of false positives or false negatives

Species	False‐negative rate	False‐positive rate
I. 10 species with the lowest false‐positive rates (following their optimal environmental conditions)		
* Alyssum granatense B. et R*.	14.286	4.26
* Alyssum scutigerum Dur*.	37.5	4.26
* Papaver hybridum*L.	18.182	4.66
* Dactylis glomerata* L.	45.455	5.62
* Aristida pungens Desf*.	37.5	6.70
* Eruca vesicaria (*L*.) Gar*.	53.846	6.81
* Arnebia decumbens(Vent.) Coss. et Kral*.	20	8.02
* Sisymbrium coronopifolium Desf*.	50	9.09
* Launaea nudicaulis (*L*.) Hook.f*.	61.29	9.28
* Thymus hirtus Wild*.	28	10.27
II. 10 species with the highest false‐negative rates (persisting under suboptimal environmental conditions)		
* Launaea nudicaulis (*L*.) Hook.f*.	61.29	9.28
* Eruca vesicaria (*L*.) Gar*.	53.846	6.81
* Peganum harmala* L.	52.381	17.82
* Sisymbrium coronopifolium Desf*.	50	9.09
* Stipa parvifloraDesf*.	47.826	11.65
* Lolium rigidum Gaud*.	47.059	20.13
* Dactylis glomerata* L.	45.455	5.62
* Launaea resedifolia ssp. eu‐resedifoliaM*.	44	28.76
* Roemeria hybrida(*L*.) DC*.	42.857	22.56
* Atractylis serratuloides Sieb*.	42.623	25.45
III. 10 species with low false‐negative and high false‐positive rates (species neither following their optimal nor persisting under suboptimal environments conditions “Species at risk”)		
* Salvia verbenaca ssp. clandestina (*L*.) Pugsl*.	0	72.54
* Onopordon arenarium (Desf.) Pomel*	0	68.12
* Carthamus lanatus*L.	0	65.43
* Scabiosa stellatassp. monspeliensis(Jacq.) Rouy*.	0	52.5
* Malva aegyptiaca* L.	0	50.93
* Telephium imperati* L.	0	47.43
* Brachypodium distachyum (*L*.) P.B*.	0	47.13
* Marrubium deserti de Noe*	0	43.90
* Bupleurum semicompositum*L.	0	42.23
* Atractylis humilis ssp. caespitosa (Desf.) M*.	0	40

### 3.2 Explaining false‐negative rates in annual and perennial species

**Figure 1:**
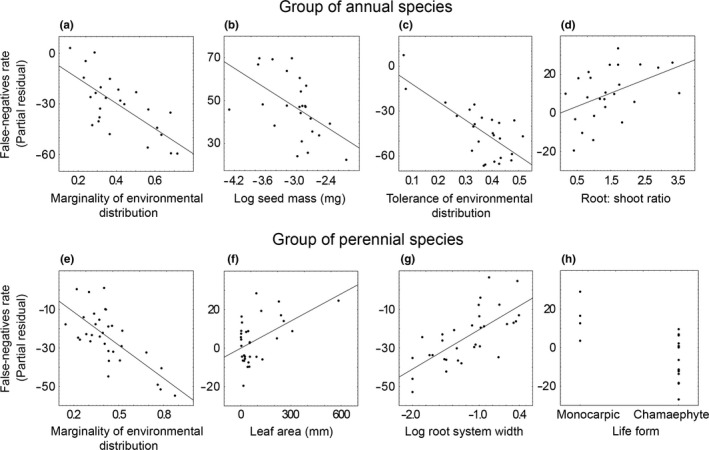
Significant relationships between false‐negative rates and species trait values. (a) marginality of environmental distribution of annual species; (b) log 10 of seed mass in annual species; (c) environmental tolerance of distribution in annual species; (d) root:shoot ratio in annual species; (e) marginality of environmental distribution in perennial species; (f) leaf area in perennial species; (j) log 10 of width of root‐system in perennial species; and (h) life form in perennial species. The figure gives false‐negative rates as partial residuals from general regression models, that is, illustrating the effect of a given trait accounting simultaneously for the other traits. For categorical variables, partial residuals are calculated separately for each category. For the full statistical results, see Table 3

False‐negative rates slightly differed between annuals and perennials (F_1,133_ = 2.92, *p *=* *.090). Within each of these groups, species traits explained 52% and 58% of the variance in false‐negative rates, respectively. False‐negative rates of annual species showed significant negative relationships with marginality of environmental distribution (Figure 1a, *p* = .014), seed mass (Figure 1b, *p* = .022), and environmental tolerance (Figure 1c, *p* = .006), and positive relations with the root:shoot ratio (Figure 1d, *p* = .058) (Table 3). These variables were selected not only by the best model but also by the nine following models (for root:shoot ratio: the seven following models Appendix S1). False‐negative rates of perennial species showed significant negative relationships with marginality of environmental distribution (Figure 1e, *p* = .0001), and positive relations with leaf area (Figure 1f, *p* = .012) and width of root‐system (Figure 1g, *p* = .001) (Table 3). A significant relationship was also found between life form and the false‐negative rate in perennial species (Figure 1h, *p* = .009). Specifically, chamaephytes had a lower false‐negative rate and monocarpic perennial species a higher false‐negative rate (Figure 1h, Table 3). These variables were selected not only by the best model but also by 10 following models (Appendix S1–S2). Four of six of the most variables controlling false‐negative ratios among either annuals or perennial species also controlled false‐negative ratios across all species pooled (Appendix S5).

**Table 3:** Summary of the regression model between species' traits and rates of false negatives and false positives (57‐60 selected species)

	Perennial species (*n* = 32–35)						Annual species (*n* = 25)					
	False‐negative rate; *R* ^*2*^ = 65%			False‐positive rate; *R* ^*2*^ = 58%			False‐negative rate; *R* ^*2*^ = 38%			False‐positive rate; *R* ^*2*^ = 52%		
	Estimate	(Std. Error)	*F*‐value	Estimate	(Std. Error)	*F*‐value	Estimate	(Std. Error)	*F*‐value	Estimate	(Std. Error)	*F*‐value
Chamaephytes	**−6.638**	**3.473**	**(5.698)+**	excl	excl	excl	NA	NA	NA	NA	NA	NA
Monocarpic	**15.456**	**4.712**	**(5.698)** **	excl	excl	excl	NA	NA	NA	NA	NA	NA
Leaf area (mm2)	**0.047**	**0.017**	**(7.322)** *	**−0.019**	**0.01**	**(3.418)+**	excl	excl	excl	0.053	0.038	(1.915)ns
Marginality of environmental distribution	**−57.162**	**12.61**	**(20.546)** ***	**excl**	**excl**	**excl**	**−74.541**	**27.823**	**(7.177)** *	**Excl**	**excl**	**excl**
Log 10 plant height (m)	excl	excl	excl	excl	excl	excl	excl	excl	excl	Excl	excl	excl
Log 10 seed mass (mg)	**−**3.858	3.281	(1.382)^ns^	**6.729**	**2.463**	**(7.462)** *	**−15.486**	**6.245**	**(6.149)** *	**15.571**	**6.704**	**(5.394)** *
Log width of root‐system (m)	**20.503**	**5.615**	**(13.329)** **	**‐11.028**	**4.416**	**(6.235)** *	excl	excl	excl	Excl	excl	excl
BRASSICACEAE membership	NA	NA	NA	NA	NA	NA	excl	excl	excl	Excl	excl	excl
Dish‐shaped Flower	excl	excl	excl	**−12.576**	**3.926**	**(4.874)** **	excl	excl	excl	**8.688**	**4.845**	**(3.152)+**
Flag‐shaped flower	excl	excl	excl	**−15.024**	**4.744**	**(4.874)** **	excl	excl	excl	Excl	excl	excl
Tube‐shaped Flower	excl	excl	excl	6.621	5.362	(4.874)^ns^	excl	excl	excl	Excl	excl	excl
Gullet‐shaped Flower	excl	excl	excl	excl	excl	excl	excl	excl	excl	**−21.017**	**7.598**	**(3.152)** *
Without obvious floral attractants	excl	excl	excl	4.589	4.255	(4.874)^ns^	excl	excl	excl	2.452	6.654	(3.152)ns
Tolerance of environmental distribution	excl	excl	excl	**29.066**	**17.12**	**(2.882)+**	**−119.693**	**39.743**	**(9.07)** **	**55.709**	**31.955**	**(3.039)+**
root: shoot ratio	excl	excl	excl	**5.629**	**1.754**	**(10.302)** **	**6.885**	**3.428**	**(4.033)+**	Excl	excl	excl
Life span	excl	excl	excl	excl	excl	excl	excl	excl	excl	Excl	excl	excl
Log root depth	excl	excl	excl	excl	excl	excl	excl	excl	excl	Excl	excl	excl
Dispersal modes	excl	excl	excl	excl	excl	excl	excl	excl	excl	Excl	excl	excl
Specific leaf area (SLA) mm²/mg	excl	excl	excl	excl	excl	excl	excl	excl	excl	Excl	excl	excl
Leaf dry‐matter content	excl	excl	excl	excl	excl	excl	excl	excl	excl	Excl	excl	excl
(LDMC) mg/g	excl	excl	excl	excl	excl	excl	excl	excl	excl	Excl	excl	excl
Corolla flower depth (mm)	excl	excl	excl	excl	excl	excl	excl	excl	excl	Excl	excl	excl

The significance is given for the predictors selected for the minimal adequate model using best subset selection: Significant values are in bold with *p *≤* *.10 (+), *p *≤* *.050 (*), *p *<* *.010 (*^*^), and *p *<* *.0010 (*^**^), ns, Not significant; excl, excluded during variable selection; NA, Not applicable.

### 3.3 Explaining false‐positive rates in annual and perennial species

**Figure 2:**
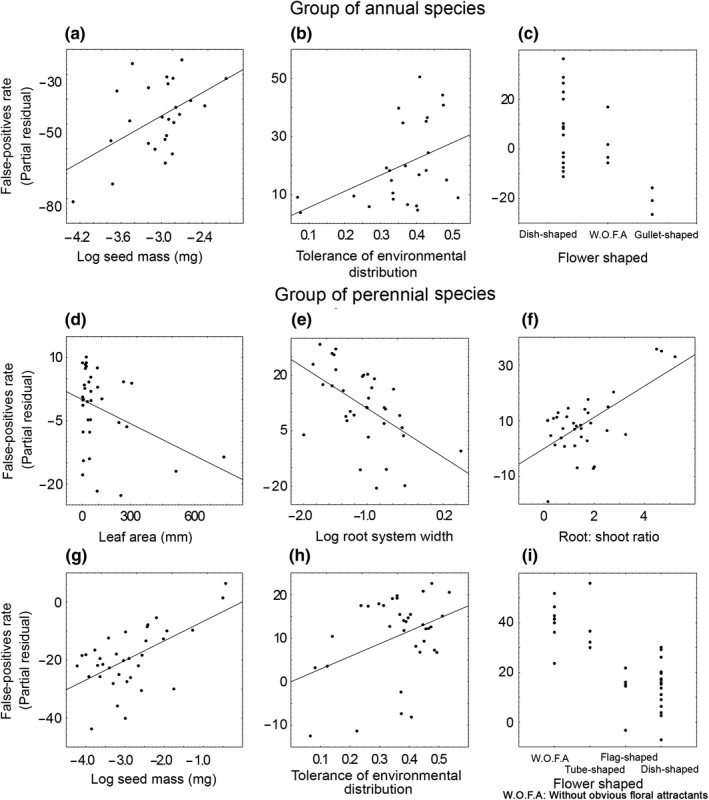
Significant relationships between false‐positive rates and species traits: (a) log 10 seed mass in annual species; (b) tolerance of environmental distribution in annual species; (c) flower shape in annual species; (d) leaf area in perennial species; (e) width of root‐system in perennial species; (f) root:shoot ratio in perennial species; (g) log 10 seed mass in perennial species; (h) tolerance of environmental distribution in perennial species; (i) flower shape in perennial species. The figure gives false‐positive rates for annual and perennial species as partial residuals from general regression models, that is, illustrating the effect of a given trait accounting simultaneously for the other traits. For categorical variables, partial residuals are calculated separately for each category

False‐positive rates did not differ between annuals and perennials (F_1,133_ = 0.11, *p *=* *.74). Within each of these groups, species traits explained, respectively, 38% and 65% of the variance in false‐positive rates. The false‐positive rates for annual species showed significant positive relationships with seed mass (Figure 2a, *p* = .032) and environmental tolerance (Figure 2b, *p* = .098) (Table 3). Furthermore, we found a significant relationship between false‐positive rates to flower shape (Figure 2c, *p* = .05), notably a positive effect for dish‐shaped flowers and a negative effect for gullet‐shaped flowers (Figure 2c Table 3). These variables were selected not only by the best model but also by nine following models (Appendix S3). False‐positive rates for perennial species showed significant negative relationships with leaf area (Figure 2d, *p* = .076), width of root‐system (Figure 2e, *p* = .019), positive relations with root:shoot ratio (Figure 2f, *p* = .003), seed mass (Figure 2g, *p* = .011), and environmental tolerance (Figure 2h, *p* = .10) (Table 3). We also found a significant relationship between false‐positive rate for perennial species and flower shape (Figure 2i, *p* = .004) notably a negative effect for dish‐shaped and flag‐shaped flowers (Figure 2i, Table 3). These variables were selected not only by the best model but also by nine following models (or six of these models in the marginally significant leaf area; Appendix S3–S4). Five of six of the similar variables controlling false‐negative ratios among either annuals or perennial species also controlled false‐negative ratios across all species pooled (Appendix S5).

### 3.4 Effect of width of root‐system on false‐positive and false‐negative rates

**Figure 3:**
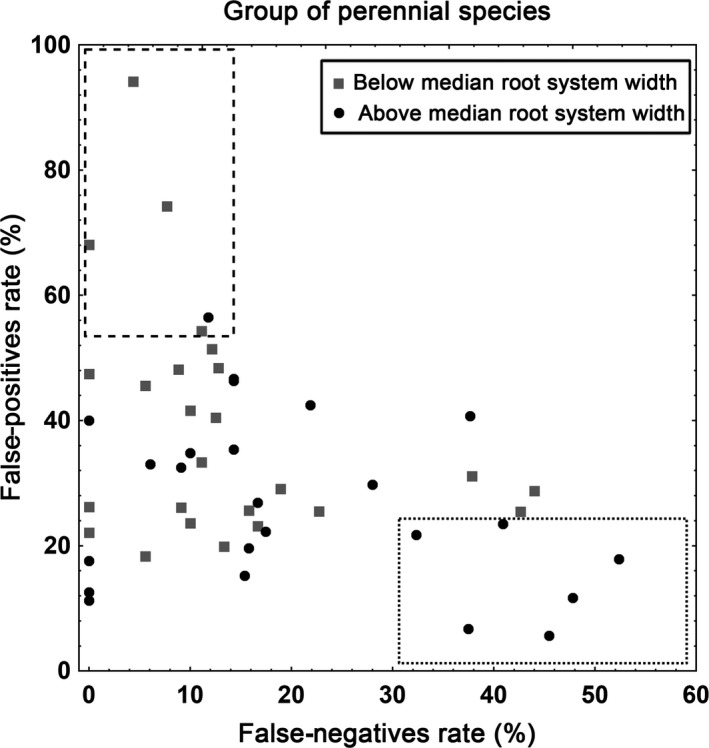
Relationships between the width of root‐system and false‐negative and false‐positive rates for perennial species. Combinations of high false‐negative rates and low false‐positive rates are delimited with dotted lines. The inverse combinations are delimited with dashed lines

The above multiple regression analyses show that explained variances are highest for perennials. The width of root‐system was the only trait significantly (*p *<* *.05) affecting both false‐positive and false‐negative rates (Table 3). The relationships are hence sufficiently strong to be illustrated without accounting for other traits (and not restricted to species for which all traits were known). We found that perennial species that have both low false‐positive rates and high false‐negative rates, that is, that track their optimal environmental conditions and occur in suboptimal conditions, have larger‐than‐median width of root‐system (Figure 3). Inversely, species that had both low false‐negative rates and high false‐positive rates, almost all had narrow root‐systems.

## 4 DISCUSSION

Are species capacities to follow their optimal environments conditions or to persist under suboptimal conditions determined by trait values? We found that traits indeed explained a high portion of the variance in these capacities (38%–52% for annuals and 58%–65% for perennials, respectively). Our results are robust across the best models, and consistent among analyses across all species pooled and analyses within perennials and annuals. Our results are largely consistent with the few other existing studies who use traits to statistically explain why some species are poorly or well predicted by distribution models (Dobrowski et al., 2011; Guisan et al., 2007; Hanspach et al., 2010; Pöyry et al., 2008; Soininen & Luoto, 2014). Low false‐positive rates usually relate to large body size, high competitiveness, low habitat tolerance, and narrow‐ranged species (Hanspach et al., 2010, 2011). High false‐negative rates relate to short life span, slow growth rate, high habitat tolerance, and high dispersal ability (e.g., wind dispersal type), species with broad ranges and small abundances (Evangelista et al., 2008; Manel, Ceri Williams, & Ormerod, 2001). To the best of our knowledge, most of these existing studies have not considered more quantitative eco‐morphological traits (Guisan et al., 2007) and, in particular, did not consider root traits, which we found to be of major importance, possibly explaining the particularly high level of explained variance in our study. The high level of explained variance in our study might also be due to the combined use of functional traits and ecological distributions in the same models, and the use of relatively complete SDMs including environmental variables covering climate, soil, and land occupation. Several interesting outcomes have emerged from our results, and we are confident that the potential methodological limitations related to the proposed approach do not impact the validity of these results.

### 4.1 Possible limitations of our study

Firstly, we estimated species optimal environmental conditions from species actual occurrences that only imprecisely reflect the true optimum for that species. Poor inference of optima and poor models should result in poor estimates of false‐positive/negative rates and hence poor correlations of these rates to traits that control a species' capacity to follow optima (or to persist elsewhere). We admit that this source of error is a potentially important limitation in our as well as many other niche‐modeling studies. We had tried to avoid this limitation by including only species present in >30 plots. For such well‐sampled species, the true optimum can be estimated with more confidence than for poorly sampled species.

Secondly, estimates of false‐positive or false‐negative rates might be biased by the methodological and sampling issues listed in the Introduction and in Table 1 IV. Each of these possible issues should produce a specific relationship of false‐negative or false‐positive rates with a particular trait value or distributional pattern of species (Table. 1 IV). For annuals, one of these bias‐indicating relationships was found: environmentally specialized (low “tolerance”) species showed high false‐negative rates, possibly reflecting a sampling bias (specialized species have many suboptimal sites to occupy, increasing the chance to find false negatives). We accounted for this bias by including “tolerance” as a predictor, and hence, tests of other predictors were likely to be unaffected. No other bias‐indicating relationship was found, sometimes quite the opposite relationship (negative effects of marginality on false‐negative rates). Bias might have been scarce because the study area, albeit geographically small, was ecologically representative of the distribution of most species that are typical of the steppe (Kadmon, Farber, & Danin, 2003). Moreover, the restriction to species of high frequency across the study area and ≥5% coverage per plot retained species that are locally abundant and rarely overlooked (Brown, 1984; Tyre et al., 2003).

Thirdly, recent studies have emphasized the importance of considering intraspecific trait variability and not only species mean trait values (Albert et al., 2010). We found that width of root‐system and seed mass were traits with a high coefficients of variation across 135 species and which significantly explanation both false negative and false positive. We admit that both traits might also show intraspecific variability on which we do not have sufficient information. Indeed, this high intraspecific variability in traits may be key to the persistence of some populations in suboptimal environmental conditions as shifts in trait values may occur quickly as a transient response to fluctuating or harsh environmental conditions (Jung et al., 2014). Accounting for this intraspecific variability could even further increase the explained variance in future studies.

### 4.2 Established‐plant traits are important mainly in perennials—dispersal‐related traits are relatively more important in annuals

We found that annual and perennial species show both striking similarities in the relationships between capacities and trait values. Annuals and perennials are similar in that (i) the capacity to follow optimal environmental conditions is high in species with small seeds and flowers attracting specialist pollinators (gullet‐ or flag‐shaped flowers), and (ii) the capacity to persist in suboptimal environmental conditions is high in species with nonmarginal distributions. In contrast, there are also many major differences between annuals and perennials in the relationships between capacities and trait values. In perennials, trait values explain distinctly more variance in both capacities than in annuals, persistence under suboptimal environmental conditions being the best explained. Wide root‐systems and large leaves relate to high capacities of both following optimal environmental conditions and persisting. In annuals, in contrast, these traits show no effect on either of the capacities. Furthermore, some trait values that relate to only one capacity have contradictory consequences in annuals and perennials. Specifically, in perennials, dish‐shaped flowers appear to be advantageous for following optimal environmental conditions, whereas in annuals, these flower shapes appear to be disadvantageous. Finally, relatively shallow roots related to different capacities, and relationships were opposite between perennials and annuals. In annuals, relatively shallow roots appear to be advantageous for following their optimal environmental conditions, whereas in perennials, relatively deep roots appear to be advantageous for persisting under suboptimal environmental conditions. Overall, similarities between annuals and perennials in relationships between capacities and trait values suggest that both groups can develop similar strategies to follow optimal conditions and persist under suboptimal conditions. In contrast, the major differences between annuals and perennials in relationships between capacities and trait values suggest that different life forms dictate different strategies. In particular, the strategy of perennials seems to be to optimize resource acquisition (wide root‐systems, large leaves) perhaps reflecting the fact that perennials need to feed a relatively large plant even during periods when there is a scarce supply of resources. Annuals, in contrast, are small and can opportunistically use the time windows during which resource availability is highest.

### 4.3 Trait values that favor both capacities, following and persisting, are costly

Some trait values favored both capacities, following optimal environmental conditions and surviving under suboptimal conditions. In perennials, these were wide root‐system and large leaves. A wide root‐system permits more efficient water uptake and hence has a dual function: It increases the individual's capacity to tolerate suboptimal abiotic conditions (severe droughts) and its competitiveness against neighbors within optimal environmental conditions (Robbins & Dinneny, 2015). Provided sufficient water uptake, large leaf area can contribute to efficient resource acquisition, both in suboptimal environmental conditions, and in optimal environmental conditions (under strong competition pressure). In annuals, small seeds favored both capacities. Small seeds might indeed provide a dual function: due to their undirected dispersal, they may frequently end up in any environmental conditions, including suboptimal ones, and due to their wide unassisted dispersal, small seeds may help to colonize distant patches of optimal environmental conditions, notably in the absence of reliable animal vectors (Jung, Böhning‐Gaese, & Prinzing, 2008). However, all these traits come at a cost. A wide root‐system requires the production of a large amount of nonphotosynthetic, and large leaf surfaces may require disproportionately greater protection against wind and desiccation than small leaves (Larcher, 2003). Small seeds, in turn, might imply a fitness cost due to the reduced competitiveness of the seedlings they produce (Grime, 1977).

### 4.4 Trait values that favor only one capacity, following or persisting, may result in a trade‐off between both

Some trait values only favored the capacity to follow optimal environmental conditions without favoring the capacity to persist in suboptimal conditions. For perennials, low root:shoot ratios are related to the capacity to follow optimal environmental conditions. In accordance with Tilman (1988), this means that species that manage to best follow their optimal conditions allocate more to above:ground biomass, thus increasing their competitiveness for light. For perennials, small seeds also only improved their capacity to follow their optimal environmental conditions. This might be due to the fact that smaller seeds are dispersed further and can reach a greater variety of conditions including optimal ones, by chance. Finally, and consistently among perennials and annuals, floral characters are only important for the capacity to follow optimal environmental conditions. Floral characters attracting specialized, efficient pollinators (Castro‐Urgal & Traveset, 2016) are advantageous only where such specialized pollinators are present, which is likely to be the optimal environmental conditions where that species is relatively abundant (Bosch, Retana, & Cerdà, 1997). Conversely, some trait values favored the capacity to persist under suboptimal environmental conditions without favoring the capacity to follow optimal conditions. In annuals, high root: shoot ratios were associated with a high capacity to succeed in suboptimal environmental conditions. In accordance with Tilman (1988), this means they allocate more to belowground biomass in order to better capture water and nutrients which are scarce in suboptimal conditions. Overall, the fact that capacities to follow optimal conditions or to persist under suboptimal conditions depend on different (and sometimes opposite) trait values potentially results in a trade‐off between these capacities.

### 4.5 Species incapable of following or persisting are at risk

The present study makes it possible to identify species that are at risk under future environmental change based on a new approach. At‐risk species are those that succeed neither in following their optimal environmental conditions, nor in persisting under suboptimal conditions. This approach differs from the conventional approach, which consists of predicting species distributions under future environmental conditions, but does not account for species' ability to follow optimal conditions or to persist in suboptimal ones (Pouget, 1979). Species that “neither follow nor persist” are listed in Table 2 III and are typically species from ephemeral patches of wind‐accumulated sand (Achour et al., 1983). These patches are indeed very hard to follow, and require extreme physiological adaptations, making establishment in other environmental conditions difficult (Pouget, 1979). These species would have escaped our attention had we only looked at their preferred environmental conditions, namely sandy habitats and high drought‐tolerance, conditions that per se will become more abundant due to future environmental change. It is interesting that some of these species have succeeded to be naturalized or to be weedy in other parts of the world, notably *Salvia verbenaca ssp. clandestina; Onopordon arenarium;* and *Carthamus lanatus* ((Born & Böcher 1998), e.g., from Australian Plant Census https://biodiversity.org.au/nsl/services/apc database). A possible explanation is that the optimal, postcultural habitats of these species are spreading by the extension of crops to the detriment of steppe vegetation and that the species profit from this spread, despite the limited capacities of following preferred environments. This present naturalization of species is not necessarily contradictory with future risk once the preferred environments start to shift or disappear, given the poor capacities of species to follow optimal environments or to tolerate suboptimal ones. In contrast to the before‐mentioned species, many of the species that are most successful in following their optimal environmental conditions (Table 2 I) appear in more stable vegetation types that are easier to follow such as undisturbed rangelands, stable dunes, or persistent salt accumulations (Pouget, 1979). Finally, many of the species that are most successful in persisting under suboptimal environmental conditions (Table 2 II) are often found persisting after an anthropic action (Achour et al., 1983; Pouget, 1979), such as temporal irrigation and fertilization (Pouget, 1979).

### 4.6 Biogeographic and evolutionary implications

We see two major implications from our study, biogeographic and evolutionary. Firstly, it seems conceivable that within a region, the capacities of species to follow their optimal, or persist under suboptimal, conditions might depend on traits that also determine the way species expand across the planet. In fact, our results are broadly consistent with the numerous studies relating species traits to their capacity to expand into a new geographic region through human introduction. Our results on species successfully following their optimal environmental conditions are consistent with the findings from many studies that successfully introduced species are characterized by traits conferring competitiveness such as high specific leaf area, low root:shoot ratio, high growth rate, or large size (Brown, 1984; Evangelista et al., 2008). Most of these introduced species, however, establish in anthropogenic, resource‐rich habitats (Brown, 1984). In resource‐poor habitats, in contrast, successfully introduced species may be characterized by high root biomass and a high water‐use efficiency or by short life cycle (Albert et al., 2010; Tyre et al., 2003), which is consistent with many of our results on species capable of persisting under suboptimal environmental conditions. The capacities of species to persist under suboptimal conditions might depend on traits that also determine the responses of species to climate change. Our results on species capable of persisting under suboptimal environmental conditions are in accordance with one study that explains range expansion of species under climate change by traits linked to dispersion, competitiveness, or habitat breadth such as large dispersal distance and seed persistence (Estrada et al., 2015).

Secondly, trait values that favor different capacities might expose species to different types of selection pressures triggering different evolutionary trajectories. By definition, the capacity to follow their optimal environmental conditions exposes species to a single constant, that is, stabilizing selection pressure (Hansen & Houle, 2004). In contrast, the capacity to persist under suboptimal environmental conditions exposes species to multiple directional selection pressures and hence to diversifying selection (Jump & Peñuelas, 2005; Savolainen et al., 2011). As both capacities depend largely on different trait values, trait values might ultimately control the type of selection in operation. Type of selection, in turn, might affect the evolutionary trajectory: stabilizing selection might favor trait conservatism within lineages (Ackerly, 2003; Jones et al., 2013; Pigliucci & Preston, 2004) while diversifying selection increases variability within species and thereby their potential to respond to environmental changes (Bussotti, Pollastrini, Holland, & Brüggemann, 2014; Grenier, Barre, & Litrico, 2016). These hypotheses on how traits favoring different capacities affect selection and ultimately evolutionary trajectories need to be tested in the future.

## 5 CONCLUSION

Prediction errors in SDMs relate strongly to species trait values, and these relationships are mostly inconsistent with what we would expect to see whether the prediction errors were mainly due to methodological problems. Instead, these prediction errors appear to mainly reflect the capacity of species to follow their optimal environmental conditions or to persist in suboptimal conditions. For a given life form, both capacities can be favored by the same trait values: among perennials by traits related to competitiveness and resource acquisition, and among annuals by traits related to wide undirected dispersal. But these all‐purpose trait values are costly. In contrast, traits related to competition, pollination, or monocarpic growth appear to be much more specific in the role they play, and trade‐offs between capacities to follow and to persist. Our results imply that species that neither succeed in following their optimal environmental conditions nor in persisting under suboptimal conditions might be particularly vulnerable to future environmental change and deserve particularly strong conservation efforts. Moreover, species trait values might ultimately control whether a species is under stabilizing selection by optimal environments or under diversifying selection by suboptimal environments, both within and across regions.

## AUTHOR CONTRIBUTIONS

HAM, LK, and AP conceived the concept; HAM and RB collected the field data with the help of LK. HAM, CHA, and AP analyzed the data. HAM, AP, and CA wrote the manuscript with the help of LK.

## CONFLICT OF INTEREST

None declared.
