# Supplementary figures and images for: How do steppe plants follow their optimal environmental conditions or persist under suboptimal conditions? The differing strategies of annuals and perennials
Source: Ecol Evol. 2017 Nov 23;8(1):135–49. doi: 10.1002/ece3.3664 (PMC5756872; doi:10.1002/ece3.3664)

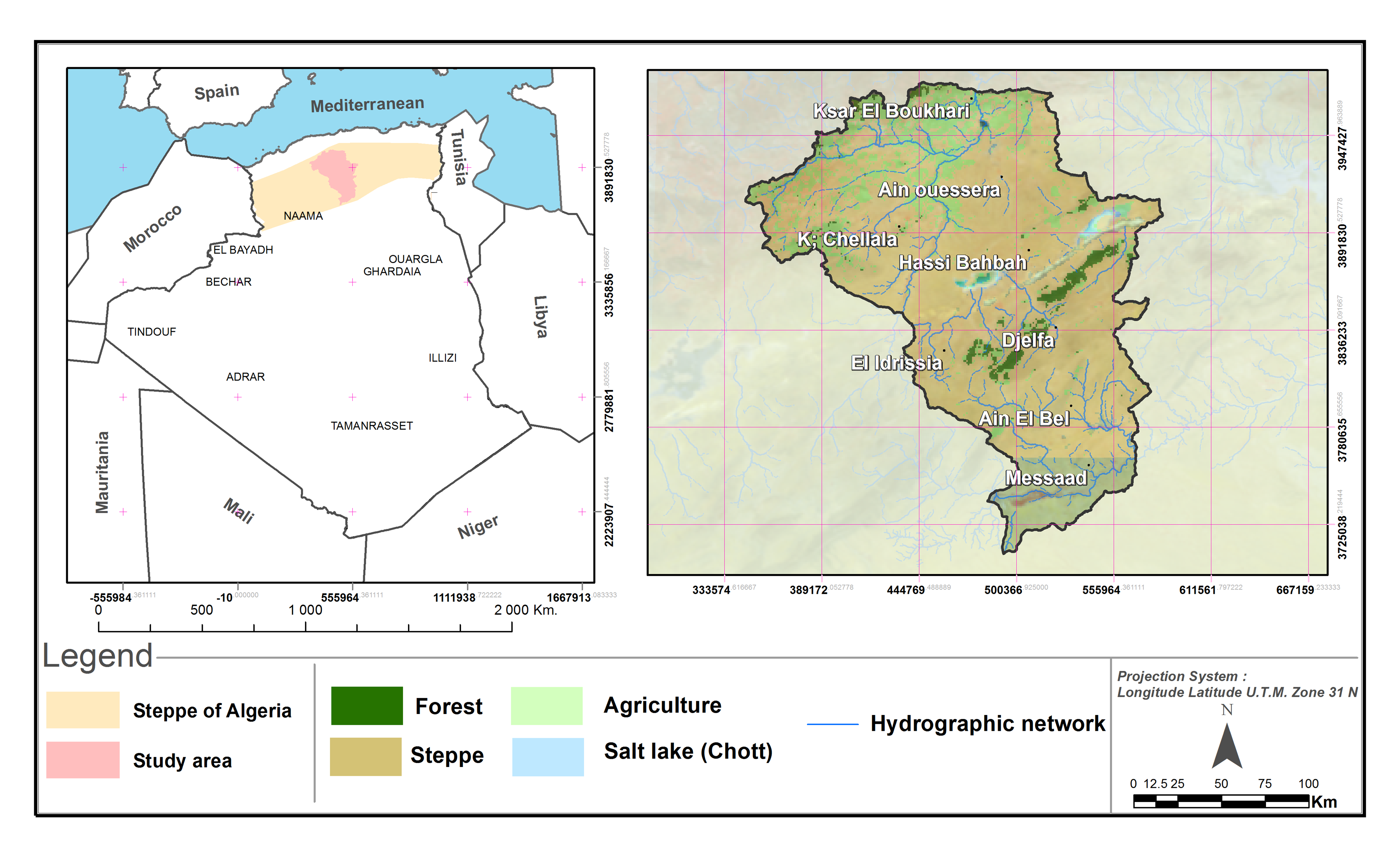

Supplement: Supplementary file 1 [file ECE3-8-135-s001.tif]
